# Low Range of Shoulders Horizontal Abduction Predisposes for Shoulder Pain in Competitive Young Swimmers

**DOI:** 10.3389/fpsyg.2019.00478

**Published:** 2019-03-06

**Authors:** Antonio Cejudo, Sheila Sánchez-Castillo, Pilar Sainz de Baranda, Juan Carlos Gámez, Fernando Santonja-Medina

**Affiliations:** ^1^Department of Physical Activity and Sport, Regional Campus of International Excellence “Campus Mare Nostrum”, Faculty of Sport Science, University of Murcia, Murcia, Spain; ^2^Department of Electronic and Computer Engineering, Higher Polytechnic School of Córdoba, University of Córdoba, Córdoba, Spain; ^3^Department of Orthopaedic Surgery, Pediatrics, Obstetrics and Gynecology, Faculty of Medicine, Hospital Universitario Virgen de la Arrixaca, University of Murcia, Murcia, Spain

**Keywords:** flexibility, injury, overhead movement, sport, pain

## Abstract

The prevalence of shoulder pain (SP) among competitive swimmers is high, and may profoundly restrict their ability to compete. This prospective cohort study investigated the association between 3 blocks of performance factors (anthropometric characteristics, sport experience and training regimen) and the presence of SP. The aims of the present study were: (a): to determine the profile of shoulder flexibility in young swimmers, (b) to analyze whether a restricted range of movement (ROM) could be a predictor of subsequent SP in young swimmers. 24 competitive young swimmers were measured in the 2016 pre-season. Measures of passive maximal shoulder extension (SE), flexion (SF), horizontal abduction (SHAB), abduction (SAB), horizontal adduction (SHADD), external (SER) and internal (SIR) rotation ROMs were taken. SP was prospectively monitored during the subsequent season using questionnaires. The data was analyzed via a binary logistic regression and ROC curves were calculated. At the follow-up, 16 swimmers (50%) had developed unilateral SP. Only reduced SHAB ROM was associated with SP [SP group 36.6° vs. pain-free group 41.5°; *p* = 0.005, *d* = -0.96 (moderate effect sizes)]. Using the coordinates of the curves, the angle of SHAB ROM that most accurately identified individuals at risk of developing SP was determined to be 39° (sensibility 0.656 and 0.375 specificity). Swimmers with limited ROM (≤39°) have 3.6 times higher risk of developing SP than swimmers with normal ROM (>39°). This study clearly shows that low range of SHAB is a risk factor for developing SP in competitive young swimmers. In the studied data, a SHAB range of 39° was found to be the most appropriate cut-off point for prognostic screening.

## Introduction

Shoulder pain (SP) has been described as the most common musculoskeletal disorder in competitive swimmers (Wanivenhaus et al., 2012) causing an impact on training, competition and swimming goals (McLaine et al., 2018). In several cohort studies, SP prevalence in swimmers is high, and may range from 40 to 91% depending on the age group and definition (McMaster, 1999; Bak, 2010; Sein et al., 2010).

Competitive swimming often train 11 months of the year (Hibberd et al., 2016) and swin over 20 h per week distributed between 5 and 7 days (Sein et al., 2010; Tate et al., 2012). Furthermore, the training intensity during the practices is quite high (Harrington et al., 2014; Ristolainen et al., 2014).

The average swimmer swims approximately 42000 to 110000 m per week depending on the competitive level (recreational swimmers vs. international swimmers) (Bradley et al., 2016). With an average stroke count of 10 complete strokes per 25 m lap (Heinlein and Cosgarea, 2010) this can equate to an average of 16800–44000 rotations of each shoulder per week.

The shoulders and upper extremities represent nearly 90% of the propulsive power for all four main strokes (Heinlein and Cosgarea, 2010). The percentage of propulsive power of the stroke comes 70–85% from the pull and 20–30% from the kick and these percentages differ according to the swimming technique (King, 1995). Swimming requires several different shoulder motions, most being performed in clockwise and counter-clockwise directions with varying degrees of internal and external rotation and scapular protraction and retraction (McLaine et al., 2018). Shoulder extension, adduction and internal rotation movements are relevant and highly reproduced in the swimming technique during the propulsive phases of the different strokes (Wanivenhaus et al., 2012).

Highly repetitive overhead loading, high volume of training and years of swimming experience places tremendous stress and adaptations on the shoulder girdle musculature and the glenohumeral joint complex adapts to the training demands (Heinlein and Cosgarea, 2010; Dischler et al., 2017; Higson et al., 2018). Swimmers have been observed as having increased thoracic kyphosis, rounded shoulders and a forward head, which can decrease subacromial-space distance (Hibberd et al., 2016; Struyf et al., 2017). In addition, demanding training programs (including swimming, strength and dry land conditioning) predisposes the swimmers shoulder to adaptive changes including decreased internal rotation and horizontal adduction ROM, and excessive external rotation with respect to no-athletes (Hibberd et al., 2016; De Martino and Rodeo, 2018).

Several studies investigated the relationship between ROM and SP in swimmers. The studies of Beach et al. (1992), Bak and Magnusson (1997), and Harrington et al. (2014) did not observe any significant relationship between SP and shoulder joint flexibility. However, Walker et al. (2012) found that swimmers with a low (<93°) external rotation ROM had an increased risk of developing shoulder pain but found no relationship between internal rotation ROM and shoulder pain; while Tate et al. (2012) found a relationship between reduced shoulder flexion, internal rotation ROM and shoulder pain in young female swimmers. Contreras-Fernández et al. (2010) found a decrease in shoulder internal and external rotation in 30 elite swimmers compared to the control group. On the contrary, Walker et al. (2012) found a relationship between SP and higher shoulder external rotation ROM (≥100°); finally, Contreras-Fernández et al. (2010) found significant differences between swimmers with or without shoulder pain in this movement. These results coincide with the systematic review of Hill et al. (2015), who reported that decreased shoulder internal rotation and either increased or decreased shoulder external rotation ROM is a risk factor for SP in swimmers.

However, no previous research has analyzed all shoulder movements in competitive swimmers in order to describe muscular and capsular adaptations of swimming (Beach et al., 1992). Sport and physical rehabilitation professionals need simple and useful tools such as reference values with “cut-off points” that classify swimmers with a normal or reduced (limited) ROM. Likewise, these professionals require reference values of the reduced ROM to help in the prevention of SP in competitive swimmers just as it has been previously determined for lower extremities in different sports injuries (Witvrouw et al., 2001; Backman and Danielson, 2011; Okamura et al., 2014; Tak et al., 2017).

Therefore, the aims of the present study were: (a): to determine the profile of shoulder flexibility in young swimmers, (b) to analyze whether restricted ROMs could be a predictor of subsequent SP in young swimmers.

## Materials and Methods

### Participants

Twenty-four young swimmers (15 males and 9 females) completed this study. All the participants were competitive swimmers (range between U12 and U20) and were recruited from three different swimming youth academies. Swimmers who volunteered for this study were accepted regardless of their current level of shoulder symptoms because swimmers frequently practice with SP.

Before data collection, participants completed a questionnaire containing questions about their sport-related background (dominant swim style, current competitive level, dominant upper extremity (defined as the participant’s preferred throw upper extremity), sport experience), demographics characteristics (age, body mass, stature and body mass index), and training regimen (weekly practice frequency, hours of swimming per week and day, resting periods, types de fitness and training load). The data from the questionnaires reported that the sample was homogeneous in potential confounding variables, except in height and years of training experience (Table 1). Besides, none of the participants were involved in systematic and specific stretching regimes in the last 6 months, apart from the 1–2 sets of 8–10 repetitions of static and dynamic stretches designated for the mains muscles of the upper extremities (e.g., pectoralis major and minor, latissimus dorsi, deltoid, trapezius, elevator scapulae and rotator cuff) that were performed daily during their pre-exercise warm-up and post-exercise cool down phases.

**Table 1:** Demographic results on swimmers participating in the study^a^.

	Males (*n* = 15)	Females (*n* = 9)	Total (*n* = 24)
Age (years)	15.7 ± 2.8	15.3 ± 0.9	15.6 ± 2.2
Body mass (kg)	61.4 ± 7.0	58.3 ± 6.5	60.3 ± 6.8
Height (cm)^∗^	172.5 ± 5.6	165.2 ± 5.7	169.8 ± 6.5
BMI (kg/m^2^)	20.6 ± 1.6	21.4 ± 2.1	20.9 ± 1.8
Years of experience swimming^∗^	7.5 ± 2.2	5.6 ± 1.5	6.8 ± 2.1
Training hours per week	15.4 ± 1.4	15.1 ± 2.1	15.3 ± 1.7


^a^Values are expressed as mean ± standard deviation. ^*^Significance statistic according to sex (p ≤ 0.002). BMI, body mass index.

The exclusion criteria for all subjects were: (1) a history of cervical or thoracic pathology; (2) previous shoulder surgery; (3) previous shoulder injury in the past 6 months; and (4) presence of SP that prevented the correct execution of the tests, including inability to achieve relaxation during testing.

The study was conducted during the season of the year 2016–2017. The measurement of the range of movement (ROM) and anthropometric measures were performed in the pre-season. At the end of the sports season, a second part questionnaire was completed by the participants about the history of SP suffered, which provided us with information regarding type of injury, location (shoulder of the dominant and non-dominant limb), frequency (number of times), severity (days of absence to training or competition), moment (training or competition) and clinical treatment (yes or no). The shoulder injury definition used was based on previous research and defined *a priori* as significant interfering shoulder pain that interfered with training or competition, or progression in training and caused cessation or modification of training or racing (McMaster et al., 1998). Questionnaires were completed with the collaboration of the parents and the coach.

Before any participation, experimental procedures and potential risks were fully explained to both parents and children in verbal and written form, and written informed consent was obtained from the parents of all participants. This study respected the ethical principles established by the UNESCO Declaration on Bioethics and Human Rights. The experimental procedures used in this study were in accordance with the Declaration of Helsinki and were approved by the Ethics and Scientific Committee of the University of Murcia (Spain) (ID: 1702/2017).

### Testing Procedure

**FIGURE 1:**
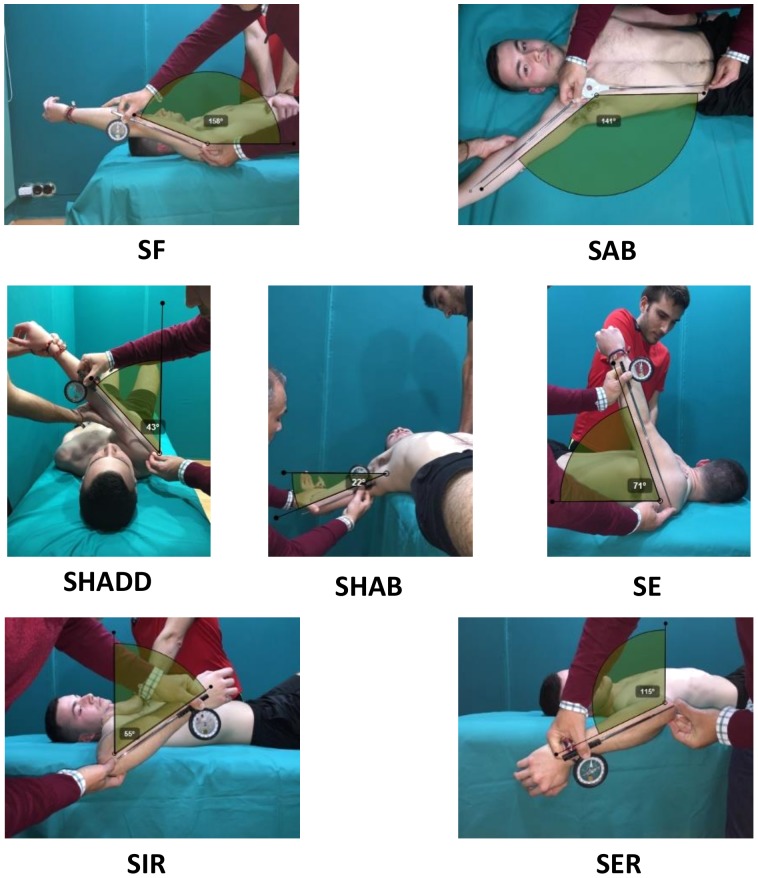
Shoulder ranges of motion of the “ROM-SPORT” protocol: SF, shoulder flexion; SAB, shoulder abduction; SHADD, shoulder horizontal adduction; SHAB, shoulder horizontal abduction; SE, shoulder extension; SIR, shoulder internal rotation; SER, shoulder external rotation. Written and informed consent has been obtained for the publication of these images from the depicted adults and from the parents/legal guardians of the minors.

The passive maximal shoulder extension (SE), flexion (SF), horizontal abduction (SHAB), abduction (SAB), horizontal adduction (SHADD), external (SER), and internal (SIR) rotation ROMs of the dominant and non-dominant upper extremity were assessed following the methodology previously described (Cejudo et al., 2015; Figure 1).

These tests were selected because they have been considered appropriate by the American Medical Association (Gerhardt et al., 2002) and included in manuals of Sports Medicine and Science (Palmer and Epler, 2002; Clarkson, 2003; Norkin and White, 2006) based on reliability and validity studies, anatomical knowledge, and extensive clinical and sport experience. In addition, previous studies have reported good to excellent to excellent intra-rater reliability for all measures (ICC: 0.85–0.99; variability: 6°–11° or less) (Mullaney et al., 2010; Cadogan et al., 2011).

*A priori* reliability was established by the primary investigator in a sample of convenience (university students; *n* = 12, ranged from age = 20–22 years) measured on two occasions, 3 day apart. The intraclass correlation coefficient (ICC) and the minimal detectable change at a 95% confidence interval (MDC_95_) values for all measures on the dominant side ranged from 0.88 to 0.96 and 3.7° to 7.2°, respectively.

One week before the start of the study, all the participants completed a familiarization session with the purpose of knowing the correct technical execution of the exploratory tests by means of the practical realization of each one of them. The dominant extremity was defined as the participant’s preferred throw upper extremity. All tests were carried out by the same two physical therapists under stable environmental conditions. Prior to the testing session, all participant performed a warm up. The overall duration of the entire warm-up was approximately 10 min (mobility of neck, shoulders, wrist, spine and pelvis, dynamic stretching by 2 sets of 15 repetitions each for the main muscles of the upper extremities each of dynamic stretches) following the considerations design by Bishop (2003), Torres et al. (2008), and Ayala et al. (2016).

After the warm-up, swimmers were instructed to perform, in a randomized order, two maximal trials of each ROM test for each joint shoulder, and the mean score for each test was used in the analyses. Swimmers were examined wearing sports clothes but without a sports shirt. The girls used a sports bra. A 30-s rest was given between trials, joint shoulder and tests.

For the measurement, an ISOMED Unilevel inclinometer (Portland, OR, United States) was used with extendable telescopic rod (Gerhardt et al., 2002), a metal goniometer with long arm (Baseline^®^ Stainless) and “lumbosant” -lumbar support- to standardize the lumbar curvature (Santonja et al., 1995). Before each assessment session, the inclinometer was calibrated to 0° with either the vertical or horizontal. The angle between the longitudinal axis of the mobilized segment was recorded (following its bisector) with the vertical (SIR, SER, and SHADD ROMs) or the horizontal (SF, SE, and SHAB ROMs) (Gerhardt et al., 2002; Cejudo et al., 2015). Regarding the assessment of shoulder abduction movement (SAB), a metal goniometer with a long arm (Baseline^®^ Stainless) was used. One or both of the following criteria determined the end-point for each test: (a) an examiner palpated or appreciated some compensation movement that increased the ROM, and/or (b) the participant felt a strong but tolerable stretch, slightly before the occurrence of pain (Cejudo et al., 2015).

### Statistical Analysis

Prior to the statistical analysis, the distribution of the raw data sets was checked using the Kolomogorov–Smirnov and Levene tests to determine normal distribution and homoscedasticity, respectively. The results demonstrated that not all data had a normal distribution nor homoscedastic (normal distribution, *p* > 0.05: SE, SF, SHAB, and SIR, not normal distribution, *p* < 0.05: SHADD, SAB and SER). Descriptive statistics including means and standard deviations were calculated for all characteristics and ROM measures separately by the variable pain (shoulder pain-free versus SP versus total) (Table 2).

**Table 2:** Results of variables for the 24 young swimmers (48 shoulders) who developed shoulder pain and those who did not (control).

Variable	Shoulder with Pain^†^ (*n* = 16)	Shoulder pain-free^†^ (*n* = 32)	Total^†^ (*n* = 48)	*p*-value	Effect sizes Cohen’s *d*
Age (years)	15.8 ± 1.9	15.4 ± 2.3	15.6 ± 2.2	0.222	Trivial (*d* = 0.18)
Body mass (kg)	61.5 ± 7.8	59.6 ± 6.2	60.3 ± 6.8	0.405	Small (*d* = 0.28)
Height (cm)	169.3 ± 8.5	170.0 ± 5.3	169.8 ± 6.5	0.860	Trivial (*d* = -0.10)
BMI (kg/m^2^)	21.4 ± 1.9	20.6 ± 1.7	20.9 ± 1.8	0.120	Small (*d* = 0.45)
Years of experience	6.0 ± 1.2	7.1 ± 2.3	6.8 ± 2.1	0.131	Medium (*d* = -0.54)
Training hours per week	15.6 ± 1.2	15.1 ± 1.8	15.3 ± 1.7	0.773	Small (*d* = 0.30)
SE (grade)**^a^**	88.3 ± 11.1	92.8 ± 8.4	91.3 ± 9.5	0.102	Small (*d* = -0.48)
SF (grade)**^a^**	174.8 ± 12.6	180.8 ± 8.7	178.8 ± 10.4	0.070	Small (*d* = -0.59)
SHAB (grade)**^a^**^∗^	36.6 ± 4.1	41.5 ± 5.5	39.8 ± 5.5	0.005	Moderate (*d* = -0.96)
SAB (grade)**^b^**	176.0 ± 9.4	176.9 ± 8.1	176.6 ± 8.4	0.722	Trivial (*d* = -0.16)
SHADD (grade)**^b^**	152.2 ± 9.2	155.1 ± 5.6	154.1 ± 7.1	0.268	Small (*d* = -0.41)
SIR (grade)**^a^**	69.3 ± 11.8	75.0 ± 12.6	73.1 ± 12.5	0.120	Small (*d* = -0.46)
SER (grade)**^b^**	129.1 ± 13.1	132.8 ± 11.9	131.6 ± 12.3	0.326	Small (*d* = -0.30)


*^†^Values are expressed as mean ± standard deviation BMI, body mass index; SE, shoulder extension; SF, shoulder flexion; SHAB, shoulder horizontal abduction; SAB, shoulder abduction; SHADD, shoulder horizontal adduction; SIR, shoulder internal rotation; SER, shoulder external rotation. ^∗^significant at p ≤ 0.05 independent-samples t-test^a^ or non-parametric Mann–Whitney U-test^b^; The magnitude of the effect size of the pooled Standardized mean differences (SMD) was interpreted as trivial or no effect if SMD < 0.2; small if SMD 0.2 to 0.59; moderate if SMD 0.6 to 1.19; large if SMD 1.20 to 2.00; very large if SMD 2.00 to 3.99 and extremely large if SMD greater than 4.00.*

To examine the existence of asymmetry of ROM between the values of the dominant and non-dominant sides, either the Student’s *t*-test (if the distribution of the data were normal) or the Wilcoxon test (if no normal distribution of the data was obtained) was used.

To examine possible differences in demographic variables and shoulder ROMs between the male and female groups for each movement, either the Independent *t*-Test (if the distribution of the data were normal) or the Mann–Whitney *U*-test (if no normal distribution of the data was obtained) was conducted.

To examine possible differences in continuous variables (anthropometric characteristics, sport-related background and training regimen variables, and 7 ROM assessments) between the shoulder pain-free and SP groups, we used either the Student’s *t*-test (if the distribution of the data were normal) or the Mann–Whitney *U*-test (if no normal distribution of the data was obtained). Univariate analyses (independent *t*-tests) were performed to compare the continuous variables between the swimmers who did and did not have SP when there was a normal distribution. A non-parametric Mann–Whitney *U*-test was performed when the distribution did not meet the criterion of normality (age, height, years of experience, training hours per week, SAB, SHADD, and SEH). Additionally, Cohen’s effect size was calculated for all results, and the magnitudes of the effect were interpreted according to the criteria of Hopkins et al. (2009), in which the effect sizes less than 0.2, from 0.2 to 0.59, from 0.6 to 1.19, from 1.20 to 2.00, from 2.00 to 3.99 and greater than 4.00 were regarded as trivial, small, moderate, large, very large and extremely large, respectively. The authors arbitrarily chose moderate as the minimal relevant effect level with practical application in the results.

The relationship between the independent variables and the dependent variable was examined by a backward stepwise binary logistic regression (Forward Selection (Conditional), inclusion probability *p* ≤ 0.05, elimination probability *p* ≤ 0.10) with OR analysis been used as in previous studies (Witvrouw et al., 2001; Fousekis et al., 2011) for estimating the simultaneous effects of several predictors instead of relative risk estimates (Hosmer and Lemeshow, 1989). Effect sizes for the OR were defined as follows: small effect OR = 1–1.25, medium effect OR = 1.25–2 and large effect OR ≥ 2 (Coombes et al., 2010).

To determine whether it is possible to find a clinically relevant cut-off point for ROM that can be used for pointing out individuals at a high risk of developing SP, receiver operating characteristic (ROC) curves were calculated. The area under the ROC curve represents the probability that a selection based on the risk factor for a randomly chosen positive case will exceed the result for a randomly chosen negative case. The area under the curve can range from 0.5 (no accuracy) to 1.0 (perfect accuracy). If it is found to be statistically significant, it means that using the risk factor as a determinant is better than guessing. Since the ROC curve plots sensitivity against 1 minus specificity, the coordinates of the curve can be considered possible cut-off points, and the most suitable cut-off can be chosen. Among the swimmers who sustained SP, Pearson’s chi-squared test was used to examine the existence of a relationship between the range of motion classification (normal and limited) and SP.

Analysis was completed using SPSS version 20 (SPSS Inc., Chicago, IL, United States). For all analyses, significance was accepted at *p* < 0.05. Data are presented as means ± s.

## Results

During the 1-year period of the study SP was identified in 16 (50%) of the 32 shoulders analyzed. Statistical analysis showed no significant sex and asymmetry (dominant vs. non-dominant limbs) difference in the incidence of SP in this study (*p* < 0.05). Three swimmers had bilateral complaints, the same number of swimmers had unilateral pain in their dominant and non-dominant shoulder (*n* = 5).

Among the variables that were assessed before the beginning of the study, the only significant difference detected between the groups (SP vs. pain-free at follow-up) was in SHAB ROM (SP group 36.6° vs. pain-free group 41.5°; *p* = 0.005, *d* = -0.96 (moderate effect sizes). The group of swimmers with SP had a reduced range of 4.9° (Table 2).

With the stepwise logistic regression analysis, of all of the variables entered into the model (Table 2), only SHAB ROM showed a small predictor of SP occurrence in the swimmers assessed (OR = 1.225 (small); 95% CI.701 to 0.953, *p* = 0.009) (Table 3). In addition, the analysis of the frequencies showed 100% of successful cases in swimmers with SP who were categorized with limited SHAB ROM (tightness of the pectoralis major and anterior capsular contracture, cut-off < 39°) according to the present study. None of the other intrinsic factors imposed a significant relative risk for SP (*p* > 0.05).

**Table 3:** Frequencies and logistical regression results: intrinsic risk factors for shoulder pain in 24 young swimmers (*n* = 48).

	Frequencies (%)			Statistics			
Variables		Normal	Tight	Odds ratio	Standard Error	95% CI	*p*-value
Shoulders pain (16 vs. 32)		
SHAB	Shoulder pain-free	68.8	31.3	1.225	0.078	0.701 to 0.950	0.009
	Shoulder pain	37.5	62.5				


*SHAB, shoulder horizontal abduction; OR, Odds Ratio (relative risk). Tightness (high risk) establish at 39° in the present study; OR > 1: increased Odds of shoulder pain; small effect OR = 1–1.25, medium effect OR = 1.25–2 and large effect OR ≥ 2 (Coombes et al., 2010); CI, Confidence Interval.*

**FIGURE 2:**
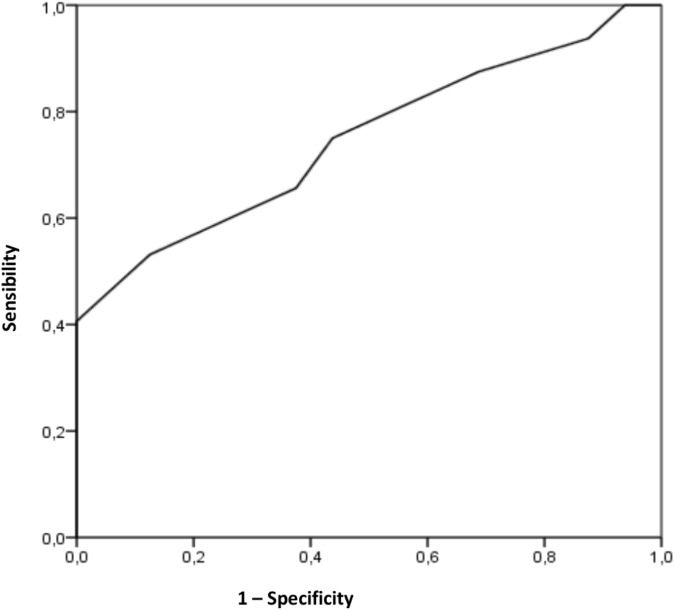
Receiver operating characteristic curve for the shoulder horizontal abduction range, as a risk factor for developing tendinopathy in the shoulder pain. The area under the curve is 0.747 (*p* = 0.006); the coordinates represent possible cut-off point in shoulder horizontal abduction range (optimal cut-off point determined to be 39°).

The area under the ROC curves was 0.747, a good predictive model accuracy (Cortes and Mohri, 2004), for the SP (Figure 2), being statistically significant (*p* = 0.006; Standard Error: 0.069; 95% Confidence Interval: 0.611 to 0.883). Using the coordinates of the curves, the angle of SHAB ROM that most accurately identified individuals at risk of developing tendinopathy was determined to be 39° (sensibility 0.656 and 0.375 specificity). Finally, the Chi-square test observed differences between the proportions of swimmers with limited and normal range in SHAB, whether or not they have SP (*p* = 0.038; 95% Confidence Interval 1.04 to 12.9). Swimmers with limited ROM (≤39°) have 3.6 times higher risk of developing SP than swimmers with normal ROM (>39°).

## Discussion

### Profile of Shoulder Flexibility

The first aim of this study was to determine the profile of shoulder flexibility in youth swimmers. It was found that the profile of shoulder flexibility in the 24 swimmers was 41.5° in the SHAB, 75° in the SIR, 92.8° in the SE, 132.8° in the SER, 155.1° in the SHADD, 176.9° in the SAB and 180.8° in the SF ROM. With respect to shoulder ROM data (SE, SER, and SHADD), this study found that swimmers with and without SP had higher values than what has been proposed for the general population (Table 4; Gerhardt, 1994; Gerhardt et al., 2002; Palmer and Epler, 2002; Clarkson, 2003; Peterson et al., 2005). It is possible that these higher values are due to musculoskeletal adaptations as a consequence of the physical-technical demands of swimming (Sainz de Baranda et al., 2015). When the results were compared with previous literature, the current study found higher ROM values than the angular values observed in 28 college swimmers, from 15 to 21 years old (SER, 47°; SIR, 105.5°; SE, 60.5; SHADD, 138.5) (Beach et al., 1992), in 8 elite swimmers from 15 to 25 years old (SER, 110°; SIR, 68°) (Bak and Magnusson, 1997), in 133 swimmers from 17 to 35 years old (SER, 110.7°; SIR, 70.6°) (Bansal et al., 2007) and in 30 elite swimmers from 15 to 25 years old (SER, 81.8°; SIR, 62°) (Contreras-Fernández et al., 2010). However, Beach et al. (1992) determined a higher range of shoulder movement than in the present study in 28 college swimmers from 15 to 21 years old (SHAB, 44°; SAB, 195.5°; SF187.5°).

**Table 4:** Comparison of shoulder range data of swimmers with normative data of the general population^†^.

	SHAB	SIR	SE	SER	SHADD	SAB	SF
General population (grade)^∗^	30–45^1,4^	65–90^2,7^	50–60^2,4^	90^2,3,7^	135^1,5^	180^2,4^	180^2,4^
Pain-free swimmers (grade)	41.5	75	92.8	132.8	155.1	176.9	180.8
Swimmers with shoulder pain (grade)	36.6	69.3	88.3	129.1	152.2	176	174.8


*SE, shoulder extension; SF, shoulder flexion; SHAB, shoulder horizontal abduction; SAB, shoulder abduction; SHADD, shoulder horizontal adduction; SIR, shoulder internal rotation; SER, shoulder external rotation. ^∗^Normative data established by 1: Gerhardt (1994); 2: Palmer and Epler (2002); 3: Gerhardt et al. (2002); 4: Clarkson (2003); 5: Peterson et al. (2005); ^†^Values are expressed in degrees.*

In the current study the ROM was similar in boys and girls and there were no significant differences when the ROM was analyzed by laterality (dominant limb vs. non-dominant limb); other studies agree with the current investigation not identifying statistically significant differences by sex (Greipp, 1985; Walker et al., 2012; Hibberd et al., 2016) and laterality (Contreras-Fernández et al., 2010; Rodeo et al., 2016; McLaine et al., 2018) in swimmers.

On the one hand, it could be possible that the similar training load per week and body mass index found in men and women are related to no differences in the ROM by sex. On the other hand, the similarity when the results were compared by laterality could be due to the symmetry in the execution of the four swimming styles (Wanivenhaus et al., 2012).

### Intrinsic Risk Factors for the Development of SP

Several factors such as age, sex, weight, height, BMI, years of competitive experience, quantity of training hours per week, competitive level or ROM have been suggested as possible risk factors for SP (Bansal et al., 2007; Tate et al., 2012; Walker et al., 2012; Harrington et al., 2014; Ristolainen et al., 2014; Struyf et al., 2017). In the current study, significant differences were found when the results were compared between sufferers and non-sufferers of SP. Concretely, those who suffered from SP had significantly less ROM than non-sufferers (*p* = 0.005; *d* = -0.96 (moderate effect sizes). Furthermore, a lower SHAB ROM (reduced extensibility of pectoralis major and anterior capsular contracture) was identified as the only predisposing factor for the manifestation of SP in this multivariable model with swimmers. The pectoralis major, teres minor, serratus anterior were the most active muscles during the initial powerful adduction, extension, neutral rotation of the humerus in the early pull-through phase (maximum forward extension to 90° flexion), as this phase is the most important in the swimmer’s propulsion (Wanivenhaus et al., 2012; De Martino and Rodeo, 2018).

The results of the present investigation are consistent with the suggestion of many experts in sports medicine who believe that muscular flexibility plays an important role in overuse injuries which includes subacromial impingement, rotator tendinosis, and biceps tendinosis (Bradley et al., 2016; Hibberd et al., 2016; Rodeo et al., 2016). However, few prospective studies have been performed to examine the shoulder joint ROM and its relationship with shoulder injuries or SP. In addition, it is worth noting that most of these studies have only analyzed the shoulder rotation ROM.

Walker et al. (2012) in their prospective study found that a decreased SIR ROM and an increased SER ROM was significantly associated with SP in competitive swimmers. Bansal et al. (2007) observed the same results in a descriptive study which compared swimmers with shoulder impingement pain with asymptomatic swimmers. Greipp (1985) determined a strong correlation between limited SE ROM and the incidence of “swimmer’s shoulder” in College swimmers. Tate et al. (2012) showed in their cross-sectional study that decreased passive SIR and SF ROM in 8–11 years old swimmers and pectoralis minor tightness in 15–19 years old swimmers were significantly associated with SP. On the contrary, four studies found no association or a low correlation between shoulder ROMs (SHAB, SIR, SE, SER, SHADD, SAB, and SF) and shoulder pain in competitive swimmers (Beach et al., 1992; Bak and Magnusson, 1997; Contreras-Fernández et al., 2010; Harrington et al., 2014).

### Determining Diagnostic Cut-Off for SHAB ROM With High Risk of Developing SP

Experts in clinical musculoskeletal assessment have established the cut-off point of limited SHAB ROM in the general adult population at 0° (Palmer and Epler, 2002; Clarkson, 2003; Peterson et al., 2005), whereas, Gerhardt (1994) and Clarkson (2003) established clinical normality in the SHAB ROM at 30° and 45° for the same population, respectively. However, these data are not the result of a prospective study but of the clinical experience of experts in musculoskeletal evaluation. In addition, no relationship between the data of normality and tightness was observed.

Walker et al. (2012) found the cut-off point in SER ROM at 93° in a longitudinal study with competitive swimmers from 11 to 27 years old. These authors identified that youth swimmers who had a low SER ROM (<93°) were more likely to develop SP. As for the present investigation, the optimal cut-off point for SHAB ROM was set at 39° in order to predict if there is a high risk of developing SP. Nevertheless, this cut-off point seems to be only suitable when it is used in youth swimmers, while it is not that applicable in other age groups, movements or sports. In addition, it has been observed that the pain of the swimmer’s shoulder depends significantly on the ROM classification at this point (limited, ≤39° and normal, >39°).

Our findings suggest that further investigation of shoulder ROM as a risk factor for the development of SP in swimmers is worthwhile. One of the principal limitations of this research was the sample size that using only U12-20 competitive swimmers means that results are generalisable to the young population; also, the registry of shoulder pain should be complemented with medical diagnostic tests to identify the specific shoulder pathology (subacromial impingement, rotator tendinosis, subscapularis tendinosis and biceps tendinosis). We propose that the investigation of shoulder ROM in combination with other factors with an artificial intelligence analysis may enhance our understanding of risk factors for shoulder pain in swimmers and provide direction for injury prevention programs.

The first strength of the present study is to define the first shoulder flexibility profile in young swimmers. Most studies evaluate two or three shoulder movements. In our case, we consider it important to assess all shoulder movement. In this way, we have identified the movement and its corresponding reference value that predisposes to shoulder pain in the swimmers analyzed. It should also be noted that a simpler, faster and more reproducible procedure has been used than the digital inclinometer and goniometer to assess the ROM. This assessment is an interesting tool for explaining to professionals in this sport.

### Possible Clinical Implications and Conclusion

These findings suggest that coaches and professionals in sport sciences should pay great attention to the regular assessment of ROM during the sport season, and consequent prescriptive measures should be taken and in order to correct the marked reduced ROM. Since reduced SHAB ROM might occur due to tightness in the pectoralis major and anterior capsular contracture (Palmer and Epler, 2002; Clarkson, 2003; Peterson et al., 2005), stretching and foam rolling are commonly used to restore ROM lost as a consequence of muscular or capsular limitations (McClure et al., 2007; Manske et al., 2010). The incidence of SP might be reduced if all swimmers with a SHAB lower than 39° improved their range of motion. Likewise, optimal shoulder ROM data will improve the physical and technical performance of swimmers in competition. A shoulder with an optimal ROM is important to perform the bilateral stroke without any assistance in body roll (Greipp, 1985; De Martino and Rodeo, 2018).

## Conclusion

This study clearly shows that a low range of shoulders horizontal abduction predisposes toward the manifestation of shoulder pain in competitive young swimmers. A shoulder horizontal abduction range of 39° was set as the optimal cut-off point in order to predict shoulder pain among young swimmers. This useful information should be employed so as to identify individuals who have higher risk in swimming teams and to enable subsequent preventive actions. The application of stretching exercises in the pectoralis major and anterior capsule of the shoulder may help to reduce shoulder pain among swimmers in the future.

## Author Contributions

All authors participated in the design, documentation, development, and writing of the manuscript. This manuscript was reviewed by all authors and all of them are responsible for its contents and providing they are responsible for the final version.

## Conflict of Interest Statement

The authors declare that the research was conducted in the absence of any commercial or financial relationships that could be construed as a potential conflict of interest.
